# THE ASSOCIATION BETWEEN WEIGHT-ADJUSTED WAIST INDEX AND COGNITIVE FUNCTION IN OLDER ADULTS

**DOI:** 10.1093/geroni/igae098.3467

**Published:** 2024-12-31

**Authors:** Xichenhui Qiu, Jiahao Kuang, Changning Wei, Xujuan Zheng

**Affiliations:** Shenzhen University, Shenzhen, Guangdong, China (People’s Republic); Shenzhen University, Shenzhen, Guangdong, China (People’s Republic); Shenzhen Polytechnic University, Foshan, Guangdong, China (People’s Republic); Shenzhen University, Shenzhen, Guangdong, China (People’s Republic)

## Abstract

**Background:**

The impact of obesity on cognitive function has generated interest, and weight-adjusted waist index (WWI) has emerged as a novel marker of obesity. However, the association between WWI and cognitive function in older adults remains unclear. This study aims to explore the relationship between WWI and cognitive performance in older adults using data from the National Health and Nutrition Examination Survey (NHANES) 2011–2014.

**Methods:**

A cross-sectional investigation was conducted using NHANES data. Multivariate regression and smoothing curve fitting were used to analyze the linear and nonlinear relationship between WWI and cognitive function, assessed by neuropsychological tests. Subgroup analysis and machine learning models were employed to evaluate the stability and associations between WWI and cognitive metrics.

**Results:**

The analysis included 3472 participants. Results showed that higher WWI was significantly associated with lower scores on cognitive tests, including CERAD-WL (learning), AFT (memory), and DSST (processing speed). This association remained stable after considering WWI as a categorical variable. The negative association was more pronounced in men and decreased with advancing age. Non-linear threshold effects were observed, with stronger correlations between WWI and cognitive function above specific thresholds. Conclusions: Increased abdominal obesity, as indicated by higher WWI, was linked to deficits in learning, memory, verbal fluency, and processing speed among older adults. These findings suggest that abdominal obesity may play a crucial role in cognitive decline in this population. Interventions targeting abdominal obesity could be important for preserving cognitive performance in older adults.

